# Promising Lipophilic PyTri Extractant for Selective Trivalent Actinide Separation from High Active Raffinate

**DOI:** 10.1021/acs.iecr.2c00104

**Published:** 2022-03-21

**Authors:** Annalisa Ossola, Eros Mossini, Elena Macerata, Walter Panzeri, Andrea Mele, Mario Mariani

**Affiliations:** † Department of Energy, Politecnico di Milano, Piazza L. da Vinci 32, I-20133 Milano, Italy; ‡ C.N.R. − Consiglio Nazionale delle Ricerche, Istituto di Scienze e Tecnologie Chimiche “G. Natta” (SCITEC), Sezione “U.O.S. Milano Politecnico”, 20133 Milano, Italy; § Department of Chemistry, Materials and Chemical Engineering “G. Natta”, Politecnico di Milano, Piazza L. da Vinci, 32, 20133 Milano, Italy

## Abstract

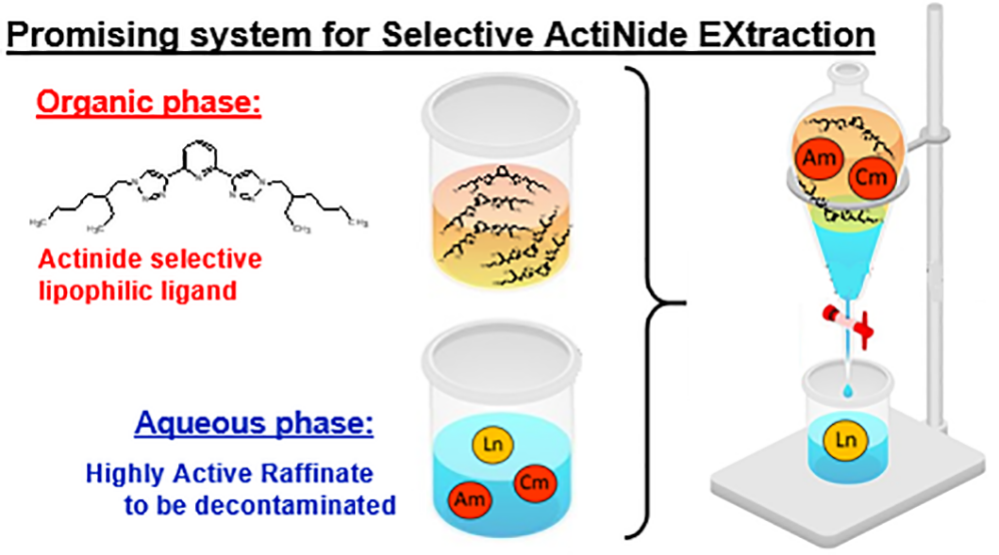

Within a spent nuclear fuel recycling strategy, in the past few years, the pyridine-bis-triazole unit was found to be rather effective and selective in minor actinide (MA) separation from synthetic high active raffinate (HAR). In this research work, the main features of the recently studied **PTEH** ligand were investigated in order to evaluate its potentialities in SANEX-like processes. Its applicability in advanced separation processes was demonstrated, even at process temperatures. It manifested satisfactory extraction properties for a successful selective An separation from Ln, easy cation release, and adequate extraction kinetics as well as outstanding hydrolytical and radiolytical stability. All the results collected in this work allowed the scientists on the committee of the H2020 GENIORS project to promote **PTEH** as a concrete alternative to the reference CyMe_4_-BTBP ligand.

## 1 Introduction

**Figure 1:**
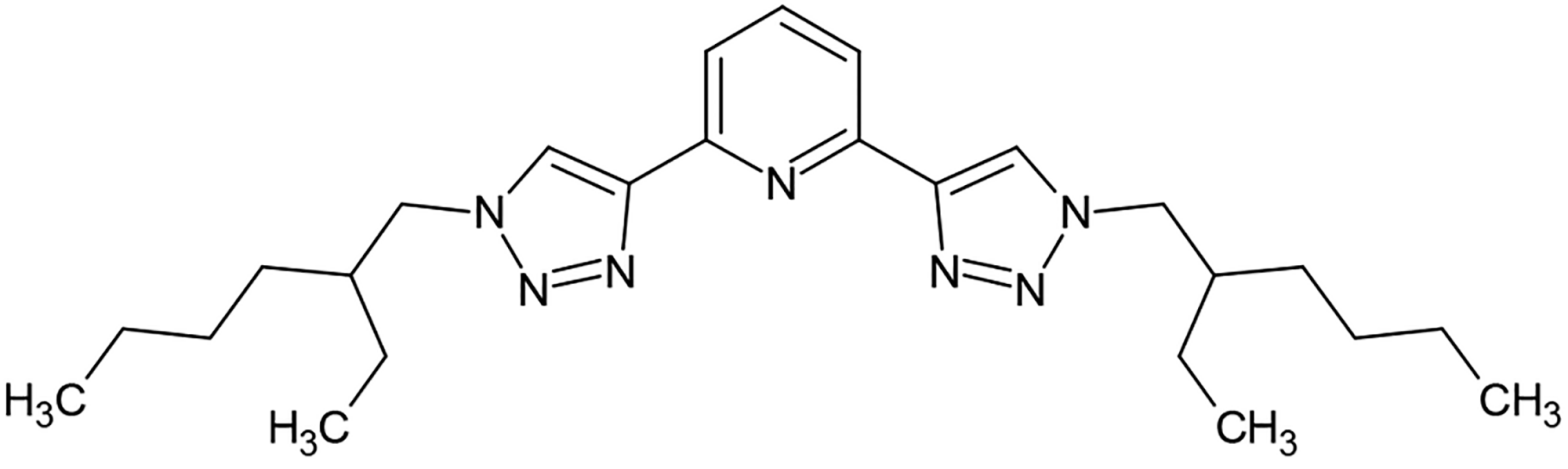
Molecular structure of PyTri-ethyl-hexyl (**PTEH**).

Recent United Nations projections disclose the population to increase to 9.5 billion by 2050 before stabilizing at around 10–12 billion by 2100. Consequently, the global energy demand is expected to considerably increase too.^1^ Nowadays, fossil fuels still represent the main source of energy, although they are considered the major responsibility of greenhouse gas (GHG) release. From this viewpoint, nuclear power has a significant potential to contribute to GHG emissions reduction, since emissions comparable with those of renewable energy sources are generated during the entire nuclear power plant life, from construction to decommissioning, including the nuclear fuel cycle.^2^ However, the development of nuclear energy is strongly limited by production, long-term management, and disposal of high level waste (HLW).^3^ IAEA estimates that about 370000 t_HM_ of spent nuclear fuel (SNF) have been discharged since the start of nuclear power based electricity production in 1954 to the end of 2013. In addition, more than 10,000 tons are discharged every year.^4,5^ Nowadays, only uranium and plutonium are reprocessed on an industrial scale by the conventional PUREX (Plutonium and URanium EXtraction) process to produce mixed oxide (MOX) fuel. Up to 2013, about one-third of discharged SNF (12,4000 t_HM_) was already reprocessed for the production of MOX fuel to be used in light water reactors. However, this process itself produces a high active raffinate, which is currently vitrified and requires storage in deep geological repositories for 3,000–10,000 years, since it still contains long-term radiotoxic minor actinides, such as Np, Am, and Cm, and fission products (FP).^6^ In this perspective, since the 1980s, the separation of transplutonium actinides from lanthanides (Ln) and the remaining FP, in view of their subsequent use as new nuclear fuel in fast reactors, has gained significant attention.^7−9^ This approach would increase the public acceptance of nuclear energy by improving the natural resources exploitation, obtaining shorter-lived or even stable nuclides, reducing the required storage time but also the radiotoxic inventory and capacity needs of repositories, and enhancing the long-term resistance toward proliferation.^10^ During the last decades, considerable scientific and technical efforts have been dedicated to the development of processes for the recovery of MA from high level waste, and many extractants have been studied to accomplish this challenging task. Particular attention has been placed on the employment of hydrophilic or lipophilic ligands constituted only by C, H, O, and N atoms, in order to be completely incinerable without generating secondary solid waste at the end of their life.^11^ In Europe, since the early 2000s, the *regular-*Selective ActiNide EXtraction (*r-*SANEX) process has been developed to selectively separate trivalent MA from the Ln contained in the downstream of the previous DIAMEX (DIAMide EXtraction) process. Subsequently, its variant, the *1-cycle-*SANEX (*1-c-*SANEX) process, has been developed as a more compact and simplified separation process aiming at directly recovering trivalent MA from the HAR of a PUREX-like process.^12^ Many heteroaromatic nitrogen donor lipophilic ligands, such as bis-triazine-pyridine/bis-pyridine/phenantroline (BTP, BTBP, and BTPhen), have been investigated for the selective MA extraction in *r-*SANEX and *1-c-*SANEX processes. They are characterized by a remarkable MA/Ln selectivity; however, the majority suffers from kinetic or stability problems in the harsh solvent extraction conditions.^13,14^ In particular, the reference CyMe_4_-BTBP (6′-bis(5,5,8,8-tetramethyl-5,6,7,8-tetrahydro-benzo[1,2,4]triazin-3-yl)-[2,2′] bipyridine) represents the most studied of BTBP ligands and was the first *r-*SANEX extracting agent to combine high, but reversible, affinity toward trivalent MA together with satisfactory hydrolytical and radiolytical stability.^15^ However, it is also characterized by limited solubility, slow kinetics, and scarce loading capability which have been partially solved by using specific diluents and/or phase transfer catalysts, hence complicating the extracting solvent.^16−19^ In more recent years, the pyridine-bis-triazole (PyTri) chelating unit was found to be promising for the selective An separation from simulated nuclear waste.^20−23^ Among the lipophilic PyTri ligands studied, preliminary promising efficiency and selectivity data were collected for the 2,6-bis(1-(2-ethylhexyl)-1H-1,2,3-triazol-4-yl)pyridine (PyTri-ethyl-hexyl – **PTEH**) ligand, reported in Figure 1.^24^ Moreover, even if, due to the different experimental conditions employed, the properties of **PTEH** and CyMe_4_-BTBP ligands cannot be compared quantitatively, these preliminary data showed that **PTEH** could be a promising alternative to the reference CyMe_4_-BTBP extractant. The aim of this work is to further investigate **PTEH** performances, thus also demonstrating the potential advantages that **PTEH** could have over CyMe_4_-BTBP. As an example, a faster extraction kinetics is an undeniable requirement for the industrial implementation of the process and would simplify the extracting system since the addition of catalysts into the organic phase would not be necessary. Therefore, the main features of the newly synthesized **PTEH** ligand were investigated in order to evaluate its potentialities in SANEX-like processes and, eventually, promote it as a valid alternative to the current reference molecule. In particular, the extraction performances of the **PTEH** solvent were ascertained as a function of different process parameters, such as ligand concentration, temperature, and feed composition. Moreover, some insights on metal–ligand complex stoichiometry and system stability toward aging, hydrolysis, and radiolysis were gathered.

## 2 Experimental Section

### 2.1 General Methods and Chemicals

2,6-Bis(1-(2-ethylhexyl)-1H-1,2,3-triazol-4-yl)pyridine (**PTEH**) was supplied by the University of Parma (Department of Chemistry, Life Sciences and Environmental Sustainability) and synthesized according to the procedure elsewhere reported.^24^

All commercially available reagents and chemicals used in this study were analytical reagent grade and used without further purification. Kerosene (reagent grade, low odor, aliphatic fraction >95%) and 1-octanol (purity ≥99%), both supplied by SIGMA-Aldrich, were used as diluents. The organic solutions were prepared by dissolving weighed amounts of **PTEH** extractant in mixtures of kerosene + 10 vol % 1-octanol.

Nitric acid solutions were prepared by diluting concentrated nitric acid (from Fluka, ≥65% w/w) with ultrapure water (Millipore, Billerica, USA; 18.2 MΩ·cm). The stock solution of ^241^Am was supplied by Eurostandard CZ (Czech Republic), and the radiotracers ^152^Eu and ^244^Cm were supplied by CERCA-LEA (France). Hexahydrated nitrates of La, Ce, Pr, Nd, Sm, Eu, and Gd (purity from 99 to 99.99%) and YCl_3_·6H_2_O (99.8%), purchased from Sigma-Aldrich, were used to prepare simplified synthetic feed stock solutions in HNO_3_ at different concentrations.

### 2.2 Extraction Experiments

Preceding the liquid–liquid extraction tests, the organic phases were pre-equilibrated with an equal volume of nitric acid, of suitable concentration, to guarantee that the aqueous phase acidity would not change during the tests. Afterward, the tests were performed according to a standard protocol. The pre-equilibrated organic phases were contacted in closed single-use Eppendorf microtubes with an equal volume of aqueous phases containing the cations to be extracted and energetically shaken by a mixer for 1 h at controlled temperature (22 ± 1 °C), if not otherwise specified. After centrifugation for achieving complete phases separation, an aliquot of 200 μL of each phase was collected. The activity of the radiotracers in each phase was quantified by γ-spectrometry (2′′ × 2′′ NaI(Tl), Silena SNIP N MCA) exploiting the γ-lines of ^241^Am and ^152^Eu at 59.5 and 121.8 keV, respectively. Instead, following proper sample preparation by diluent evaporation to dryness on a steel planchet, the activity concentrations of ^241^Am and ^244^Cm were measured by means of α-spectrometry (ORTEC OCTÊTE PLUS alpha spectrometer) taking advantage of their main α-lines at 5.485 MeV and 5.804 MeV, respectively. In order to minimize mass balance errors, the results obtained for ^244^Cm were normalized on the activity concentrations of ^241^Am obtained from γ and α spectrometries. Instead, the concentration of stable elements (Y and Ln) was assessed by inductively coupled plasma mass spectrometry (ThermoFisher X-SeriesII ICP-MS). While the aqueous phases were directly measured after appropriate dilution with ultrapure 1 vol % HNO_3_, the organic phases were mixed with the nonionic surfactant TRITON-X-100 before dilution to a suitable cation concentration. The ICP-MS calibration solutions were purchased from INORGANIC VENTURES (Christiansburg, Virginia).

Extraction tests were considered reliable only if no third phase formation was observed and the activity balance was 100 ± 5%.

Distribution ratios, *D*_M_, were computed as the ratio between the concentration of the cations (M) in the organic phase and that in the aqueous phase. The error linked to distribution ratios between 0.01 and 100 is about ±5%, while it rises up to ±20% for values outside this range. The selectivity is described by the separation factor (*SF*An/Ln) which is the ratio of *D*_An_ over *D*_Ln_, namely *D*_An_/*D*_Ln_.

According to the procedure above-described, different types of experiments were conducted:

#### 2.2.1 Ligand Concentration Dependence

In order to evaluate the impact of **PTEH** concentration on system performances, different extraction tests were performed with variable ligand concentrations (from 0.12 to 0.20 M) dissolved into the kerosene + 10 vol % 1-octanol mixture, optimized in a previous work.^24^ The pre-equilibrated organic phases were contacted with 3 M nitric acid solutions spiked with trivalent ^241^Am, ^244^Cm, and ^152^Eu.

#### 2.2.2 Complex Stoichiometry

In order to deepen the study of **PTEH** selectivity and obtain some preliminary information regarding the stoichiometry of metal/ligand complexes formed by **PTEH**, a slope analysis has been performed on the results of ligand concentration dependence experiments (see point 1).

#### 2.2.3 Extraction Kinetics

Since the extraction kinetics of a system is a very important parameter to be evaluated in view of an application to the apparatus employed in multistage processes, such as centrifugal contactors and mixer-settlers, liquid–liquid extraction tests were performed varying the mixing time from 5 to 60 min. In particular, the mixing has been performed in microtubes by a Thermo Fisher mixer at 1100 rpm at controlled temperature. The pre-equilibrated organic phases (0.2 M **PTEH** in kerosene + 10 vol % 1-octanol) were contacted with 3 M nitric acid solutions spiked with trivalent ^241^Am and ^152^Eu.

#### 2.2.4 Selectivity toward Ln

In order to further assess the **PTEH** selectivity for MA toward the Ln series, solvent extraction tests were performed employing a more complex aqueous feed (see Table 1). The quantity and relative proportion of Y and Ln in such an aqueous feed were previously defined so as to better mimic a simplified PUREX raffinate. The pre-equilibrated organic phases (0.2 M **PTEH** in kerosene + 10 vol % 1-octanol) were contacted with 1 to 3 M nitric acid solutions (see Table 1 for composition).

**Table 1:** Composition of the Simplified Synthetic Feed Solution Used in the Liquid–Liquid Extraction Tests

element	concn [mg/L]	element	concn [mg/L]
^241^Am	traces	Pr	269
^152^Eu	traces	Nd	904
Y	85	Sm	189
La	301	Eu	36
Ce	706	Gd	63

#### 2.2.5 Temperature Dependence

With a view to obtain some information regarding the system performances at process temperatures (30 ≤ *T* ≤ 40 °C), some solvent extraction tests were carried out by varying the temperature in the 20 ≤ *T* ≤ 50 °C range. The pre-equilibrated organic phases (0.2 M **PTEH** in kerosene + 10 vol % 1-octanol) were contacted with 3 M nitric acid solutions spiked with trivalent ^241^Am, ^244^Cm, and ^152^Eu.

#### 2.2.6 Resistance toward Hydrolysis and Radiolysis

Preliminary information about the ligand radiochemical stability was obtained by performing liquid–liquid extraction tests and HPLC (high performance liquid chromatography) coupled with ESI-MS (electrospray ionization - mass spectroscopy) analyses on pre-equilibrated **PTEH** solutions irradiated up to 300 kGy by means of a ^60^Co source (2.5 kGy/h dose rate). In some cases, the organic phases were irradiated in contact with an equal amount of 3 M HNO_3_ in order to evaluate its impact on the system degradation. In order to estimate the radiolysis effect, some reference solutions were not irradiated but just aged in the dark at room temperature (i.e., 22 ± 1 °C) for the same length of time, whether in contact or not with 3 M HNO_3_. All samples were stored in the dark at 4 ± 1 °C until the irradiation at the highest absorbed dose was completed. Consequently, the same thermal treatment was guaranteed to all samples. Preceeding aging/irradiation, all organic solutions were pre-equilibrated with an equal volume of 3 M HNO_3_. The liquid–liquid extraction experiments were performed by contacting each aged and irradiated organic phase with fresh solutions of 3 M HNO_3_ spiked with trivalent ^241^Am, ^244^Cm, and ^152^Eu. The results were compared with those obtained with a fresh **PTEH** solution. The chemical composition of the irradiated samples was characterized by HPLC coupled with ESI-MS techniques. HPLC-MS analyses were performed with 1100 Series (Agilent) and Bruker Esquire 3000 PLUS instruments. The former was equipped with a Purospher STAR RP-18 end-capped column (3 μm). The latter exploits an ESI Ion Trap LC/MSn System, equipped with an ESI source and a quadrupole ion trap detector (QIT). ESI-MS acquisitions were executed on HPLC outflow, set to 0.5 mL/min (flow rate limit). HPLC-MS measurements were performed at 30 °C using an isocratic mobile phase [(A: CH_3_CN/TFA 0.1% v/v), (B: H_2_O)]. The assignment of some of the species detected was confirmed also by direct ESI-MS analysis. The *m*/*z* species of interest were isolated, fragmented, and detected (MS^2^) to ease the byproduct identification.

#### 2.2.7 Cation Release Capability

With the aim to verify the release of complexed cations, necessary both in view of organic solvent recycling and of further purification steps prior to fuel elements manufacturing, back-extraction tests were performed on loaded fresh, aged, and irradiated organic phases (0.2 M **PTEH** in kerosene + 10 vol % 1-octanol). As a matter of fact, the removal of complexed metal ions and the organic solvent recycling would improve economics and sustainability of the future reprocessing plant. Even if the ligand is selective for MA, since small amounts of Ln could be extracted anyway, it is important to verify also their release. In fact, if kept in the organic phase, they could cause problems in the long term operation of the plant. In order to keep the solvent formulations as simple as possible, namely without the introduction of new reagents, diluted nitric acid solutions were employed for cation back-extraction as it was previously proposed for other lipophilic extractants, except for the BTBPs that required the addition of glycolic acid to the aqueous stripping solution.^15^ Thus, the back-extraction tests were performed with fresh 0.1 M HNO_3_ solutions.

## 3 Results and Discussion

The promising properties of **PTEH** in kerosene/1-octanol mixtures have already been outlined and are reported in a previous work.^24^ In particular, among the lipophilic PyTri compounds formerly investigated, only **PTEH** resulted in being well soluble in all the tested diluents, especially in kerosene/1-octanol mixtures usually employed for other extractants. Its optimal working diluent mixture was found to be kerosene + 10 vol % 1-octanol. In such conditions, *D*-values of ^241^Am and ^152^Eu increase by increasing nitric acid concentration in the aqueous phase. In particular, *D*_Eu_ was always well below 1, while *D*_Am_ was under the unity at 1 M nitric acid concentration of the aqueous phase and increases up to 5.5 at 3 M nitric acid concentration of the aqueous phase. Thus, an effective separation of ^241^Am from ^152^Eu is achieved for HNO_3_ concentrations of the aqueous phase higher than 2 M. The *SF* of Am(III) over Eu(III) were found to be close to and, in some cases, even higher than 80, resembling the values found for the promising hydrophilic PyTri compounds,^20^ and for these reasons its performances have been more widely investigated in this work.

### 3.1 Ligand Concentration Dependence

**Figure 2:**
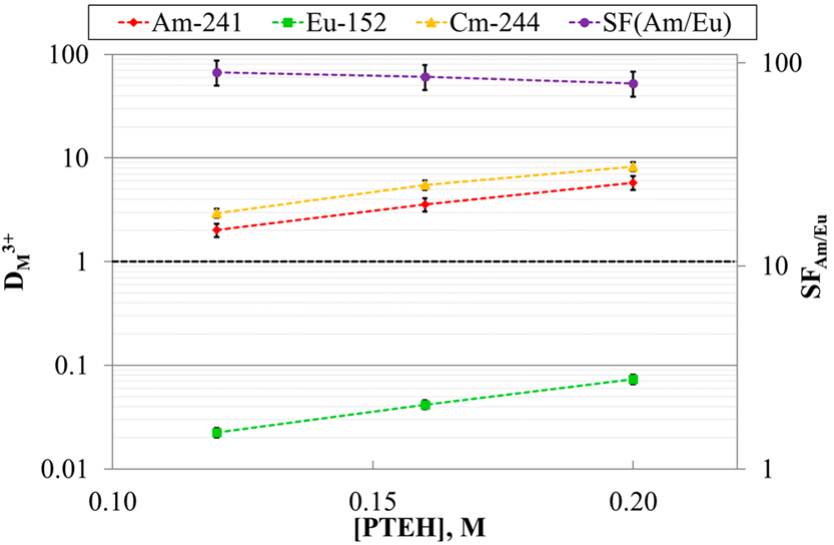
D-values and *SFs* of ^241^Am, ^244^Cm, and ^152^Eu as a function of **PTEH** concentration. Organic phase: **PTEH** in kerosene + 10 vol % 1-octanol mixture. Aqueous phase: 3 M HNO_3_ solutions spiked with trivalent ^241^Am, ^244^Cm, and ^152^Eu (ca. 10 kBq/mL each).

The distribution ratios of trivalent ^241^Am, ^244^Cm, and ^152^Eu as a function of the **PTEH** concentration are reported in Figure 2. From the data, it was possible to compute the decontamination factors, reported in Table 2, as the ratio between the activity concentration of radionuclides in the feed (i.e., aqueous phase to be decontaminated) and in the raffinate (i.e., aqueous phase decontaminated by liquid–liquid extraction). As observable, all the D-values linearly increase with increasing ligand concentration. Contrarily, Eu(III) decontamination factors are not significantly influenced by **PTEH** concentration and remain close to unity, further confirming the scarce affinity of the ligand toward Ln. Although the process requirements are successfully fulfilled in all the conditions, the employment of the highest ligand concentration, leading to the highest ^241^Am and ^244^Cm D-values, guarantees a broader safety margin toward possible system composition alteration. Interestingly, although unsatisfactory, separation factors between Cm and Am of 1.46 ± 0.21, 1.54 ± 0.22, and 1.43 ± 0.20 were obtained for ligand concentrations of 0.12, 0.16, and 0.20 M, respectively.

**Table 2:** Decontamination Factor at Increasing PTEH Ligand Concentration in the Organic Phase

	decontamination factor		
[PTEH]	0.12 M	0.16 M	0.2 M
^241^Am	3.0 ± 0.3	4.6 ± 0.5	6.8 ± 0.7
^244^Cm	3.9 ± 0.4	6.5 ± 0.7	9.3 ± 0.9
^152^Eu	1.0 ± 0.1	1.0 ± 0.1	1.1 ± 0.1

### 3.2 Complex Stoichiometry

**Figure 3:**
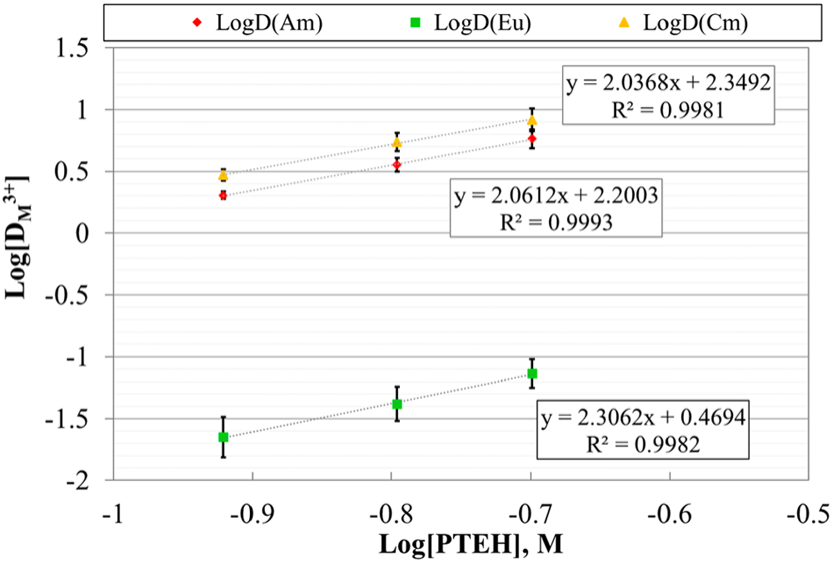
Logarithm of D-values of ^241^Am, ^244^Cm, and ^152^Eu as a function of the logarithm of **PTEH** concentration, at 22 ± 1 °C. Organic phase: **PTEH** in kerosene + 10 vol % 1-octanol. Aqueous phase: 3 M HNO_3_ solutions spiked with trivalent ^241^Am, ^244^Cm, and ^152^Eu (ca. 10 kBq/mL each).

The same data reported in Figure 2 can be processed and plotted as the logarithm of the D-values as a function of the logarithm of ligand concentration, as reported in Figure 3. This data processing approach, the so-called slope analysis, is useful to obtain preliminary information on metal/ligand complex stoichiometry.

Slopes of 2.06 ± 0.05, 2.03 ± 0.10, and 2.30 ± 0.05 were derived from the linear regression of Am(III), Cm(III), and Eu(III) data, respectively. This entails the prevailing presence of 1:2 metal/ligand complex stoichiometry. Analogous complex stoichiometry can be found in the literature for the CyMe_4_-BTBP ligand in 1-octanol, that is the reference SANEX solvent.^15^ The equilibrium reaction reported in eq 1 shows the simplified probable **PTEH** complexation with An and Ln cations.Hypothesized complexation mechanism of PTEH with trivalent cations
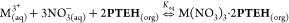
1

### 3.3 Extraction Kinetics

**Figure 4:**
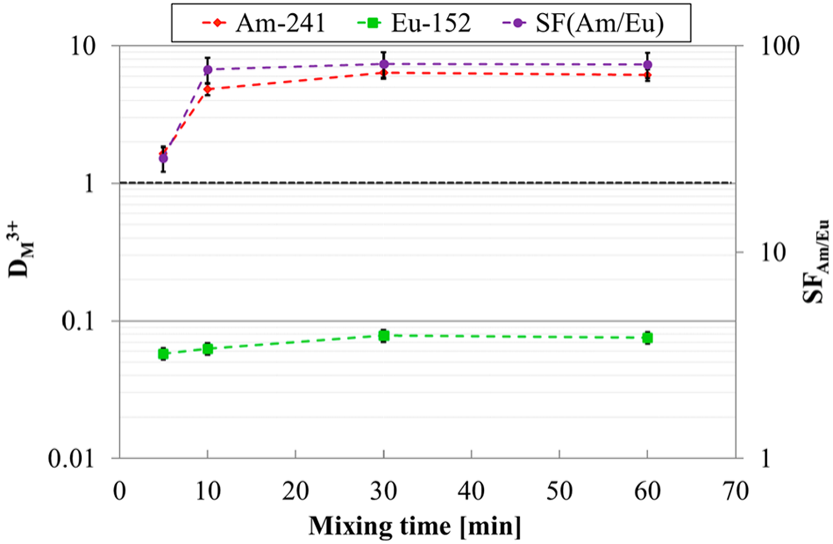
D-values and *SFs* of ^241^Am and ^152^Eu as a function of mixing time. Organic phase: 0.2 M **PTEH** in kerosene + 10 vol % 1-octanol. Aqueous phase: 3 M HNO_3_ solutions spiked with trivalent ^241^Am and ^152^Eu (ca. 10 kBq/mL each).

The aim of this experiment is evaluating the required mixing time for achieving the equilibrium. As reported in Figure 4, the equilibrium resulted in being almost reached within 10 min of mixing. Thanks to these results, all other data reported in this manuscript and obtained after 60 min of mixing are reliable, since they describe the system at equilibrium. The relatively rapid extraction kinetics for **PTEH** bodes well for its application under countercurrent solvent extraction conditions using mixer-settlers or pulsed columns, a potential benefit compared to the reference CyMe_4_-BTBP ligand which requires the employment of phase-transfer catalysts to improve its very slow extraction kinetics.^16^ The faster extraction kinetics shown by **PTEH** could ease the development of the process flow-sheet, as the flow-rates could be increased and, consequently, the number of extraction stages reduced with respect to CyMe_4_-BTBP, without hampering the system performance.

### 3.4 Selectivity toward Ln

**Figure 5:**
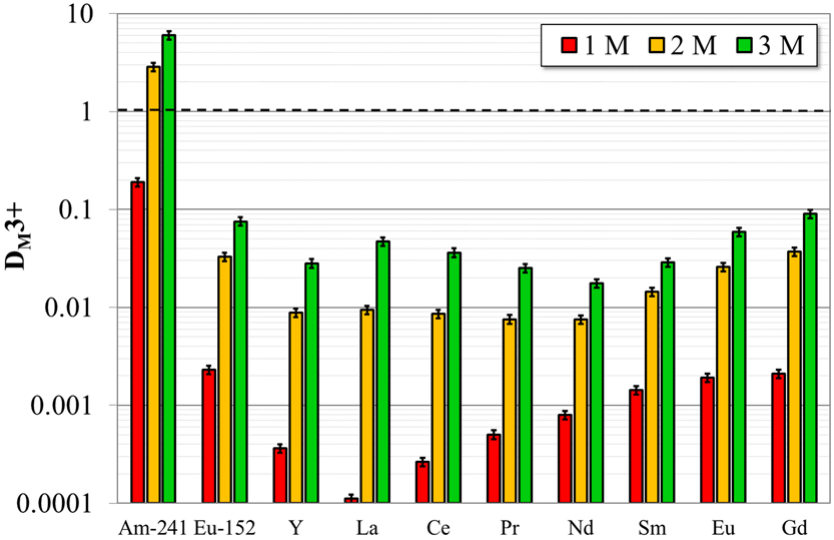
D-values of trivalent ^241^Am, ^152^Eu, Y, and lighter Ln (La–Gd) as a function of the nitric acid concentration of the aqueous phase. Organic phase: 0.2 M **PTEH** in kerosene + 10 vol % 1-octanol mixture. Aqueous phase: HNO_3_ solutions loaded with Y and lighter Ln (La–Gd) for a total concentration of about 0.02 M, besides trivalent ^241^Am and ^152^Eu as radiotracers (ca. 10 kBq/mL each)

Figure 5 shows the D-values of trivalent Am and Ln as a function of nitric acid concentration of the aqueous phase. The aim of this experiment is assessing the capability of **PTEH** of selectively extracting MA in the presence of a more complicated and representative aqueous feed. In all cases, ^152^Eu data obtained by gamma spectrometry were found to be consistent with those of stable europium obtained from mass spectrometry, even if ^152^Eu is in tracer concentration while the second is at approximately 36 mg/L. Coherently with the results obtained with radiotraced solutions,^24^ the *D*-values of all these cations increase at increasing nitric acid concentration of the aqueous phase. In particular, at 1 M nitric acid concentration of the aqueous feed, the ^241^Am *D*-value is below the unity and, thus, unsuitable for extraction of Am from the aqueous feed. Contrarily, an effective separation of ^241^Am from Y and Ln was obtained for nitric acid concentrations higher than 2 M. Promising *SF* between ^241^Am and trivalent Ln were obtained and are reported in Table S1. As an example, at 3 M nitric acid concentration of the aqueous feed, the *SF* between Am and the less and the most extracted Ln, namely Nd and Gd, are about 340 and 66, respectively.

The aqueous feed decontamination factors obtained with 0.2 M **PTEH** in kerosene + 10 vol % 1-octanol solutions, as a function of the nitric acid concentration of the aqueous phase, are displayed in Table 3. They were calculated as the ratio between the activity concentration of radionuclides, or concentration of the stable elements, in the aqueous feed and in the raffinate. As noticeable, by increasing the nitric acid concentration of the aqueous phase from 1 to 3 M, higher Am(III) decontamination factors were achieved, in agreement with results of Table 2. Conversely, in the same range, no significant changes in the ^152^Eu(III) decontamination were highlighted. In addition, the decontamination factors related to the stable Ln remain constant around approximately 1 in all the extraction conditions, implying that Ln are scarsely extracted into the organic phase.

**Table 3:** Decontamination Factors of Aqueous Feed as a Function of Nitric Acid Concentration of the Aqueous Phase

	decontamination factor		
HNO_3,eq_	1 M	2 M	3 M
^241^Am	1.2 ± 0.1	3.8 ± 0.4	7.0 ± 0.7
^152^Eu	1.0 ± 0.1	1.0 ± 0.1	1.1 ± 0.1
Ln	1.0 ± 0.1	1.0 ± 0.1	1.0 ± 0.1

### 3.5 Temperature Dependence

**Figure 6:**
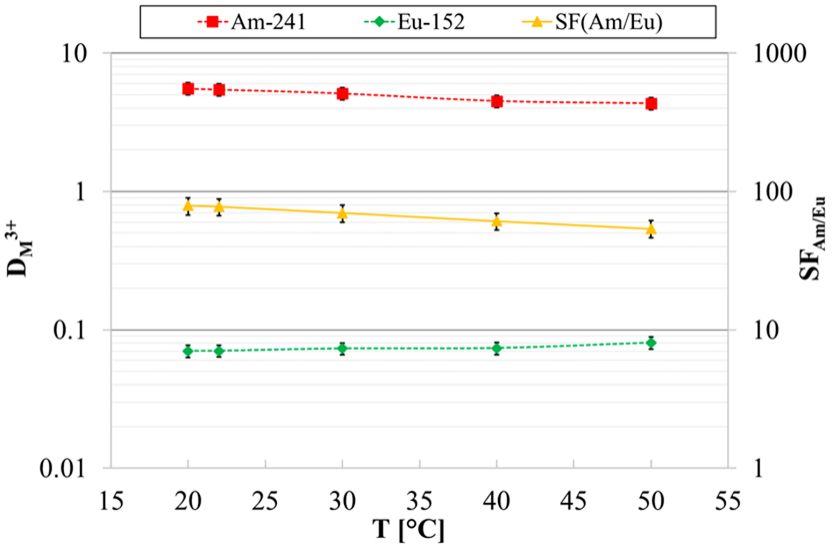
D-values of ^241^Am and ^152^Eu and SF_Am/Eu_ as a function of *T*. Organic phase: 0.2 M **PTEH** in kerosene + 10 vol % 1-octanol. Aqueous phase: 3 M HNO_3_ solutions spiked with trivalent ^241^Am and ^152^Eu (ca. 10 kBq/mL each).

Basic information about the temperature influence on system performances can be derived from the slopes of the *D* vs temperature plot (see Figure 6). This experiment provides meaningful results, since temperature fluctuations may occur in the future industrial facility and affect the extraction process.

In particular, the slopes depict the sensitivity of each extracted element toward temperature variation: the bigger the slope is, the higher the sensitivity is. Therefore, as noticeable from Figure 6, a slightly higher variation of ^241^Am distribution ratios with respect to ^152^Eu could be inferred. In fact, ^241^Am extraction slightly decreases with increasing the temperature, thus suggesting an exothermic nature of its extraction equilibria. Instead, ^152^Eu extraction appeared not to be affected by temperature. A confirmation of these different trends is given by the separation efficiency (^241^Am/^152^Eu) which decreases from 79 to 54 as the temperature increases from 20 to 50 °C, respectively. However, the system performance remains absolutely satisfactory in the whole temperature range considered.

Further studies are in progress in order to deepen and better understand the **PTEH** extraction mechanism. Additional techniques could also be exploited, such as TRLFS, UV–vis, or calorimetry.

### 3.6 Resistance toward Hydrolysis and Radiolysis

The results of solvent extraction experiments executed with irradiated and/or aged **PTEH** solutions are reported in Figure S1. Regarding aging, after pre-equilibrium, **PTEH** solutions were left to age in the dark at room temperature (22 ± 1 °C) for 71 days (see Figure S1, test *ii*). Instead, about hydrolysis, other solutions were aged for 169 days in contact with an equal volume of 3 M nitric acid (see Figure S1, test *iii*), always in the dark at room temperature (22 ± 1 °C). Concurrently, other pre-equilibrated solutions were subjected to gamma irradiation at 100, 200, and 300 kGy, not in contact (see Figure S1, tests *iv*, *v*, and *vi*, respectively) or in contact with an equal volume of 3 M nitric acid (see Figure S1, tests *vii* and *viii*). In order to ascertain the effect of radiolysis alone, the first set of tests (namely tests *iv*, *v*, and *vi*), which underwent aging and radiolysis, must be compared with test *ii*, which instead underwent only aging for the same period of time. Instead, the second set of tests (namely tests *vii* and *viii*), which underwent both hydrolysis and radiolysis, must be compared with test *iii*, which underwent only hydrolysis for the same period of time. As reference, liquid–liquid extraction tests were carried out with pre-equilibrated fresh **PTEH** solutions (see Figure S1, test *i*). As discernible, no significant variations of the extraction efficiency were pointed out for solutions subjected to aging, hydrolysis, and irradiation up to 300 kGy with respect to the results of the fresh solution. In fact, alterations of both D-values of ^241^Am, ^244^Cm, and ^152^Eu and of the corresponding *SFs* are within the experimental uncertainty. A comparable radiochemical stability has been highlighted also for the hydrophilic PyTri compound, PyTri-diol.^25^

With the aim of further confirming the promising radiochemical stability highlighted and attempting the identification of potential degradation byproducts, HPLC-MS and direct ESI-MS analyses were performed on the same fresh, aged, and irradiated **PTEH** solutions used in the solvent extraction experiments.

**Figure 7:**
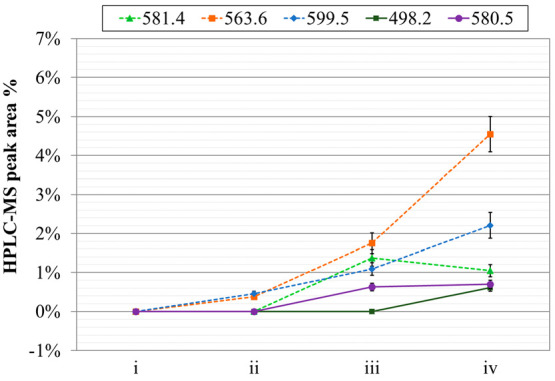
HPLC-MS peak areas of **PTEH** main byproducts (indicated in g·mol^–1^) in different **PTEH** solutions: (i) fresh, (ii) aged, (iii) irradiated at 100 kGy, and (iv) irradiated at 200 kGy.

**Figure 8:**
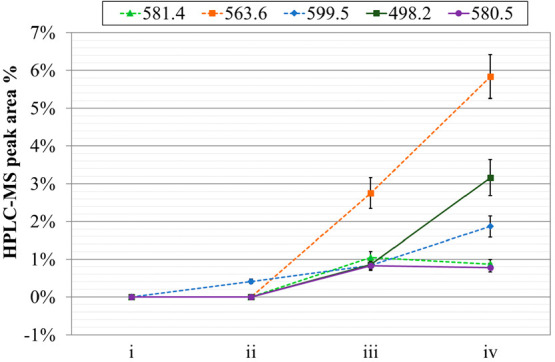
HPLC-MS peak areas of **PTEH** main byproducts (indicated in g·mol^–1^) in different **PTEH** solutions: (i) fresh, (ii) aged in contact with 3 M HNO_3_, (iii) irradiated at 100 kGy in contact with 3 M HNO_3_, and (iv) irradiated at 200 kGy in contact with 3 M HNO_3_.

These analyses allowed the identification of few new peaks in the chromatograms of the aged and irradiated samples. With the purpose of evaluating the ligand loss and following the evolution of the degradation byproducts, a postprocessing of the HPLC-MS data was performed. The ion currents of the new species were isolated and extracted as a function of the analysis time. Afterward, for each degradated species, the ratio between the ion current signal area and the total area of all signals was evaluated (see Figure 7 and Figure 8). These peaks could be related to degradation byproducts since they were absent in fresh solutions. As noticeable in Figure 7 and in Figure 8, in both cases the intensity signals of most of the species increased with aging and irradiation.

The separation and subsequent fragmentation of each byproduct, at its specific *m*/*z*, was obtained by exploiting ESI-tandem mass (MS^2^) spectrometry, after the ionization step. This allowed the proposal of some plausible hypotheses about the structure of the observed species. Consistently with the wide radiation chemistry literature^26−29^ and with the hypothesized degradation mechanism of the hydrophilic PyTri derivative,^21^ the lateral branches were supposed to be the weakest part of the ligand structure. Contrarily, the aromatic moieties were considered as the most hydrolytically and radiolytically resistant part of the ligand. Furthermore, ligand degradation is expected to be mainly attributable to indirect radiolysis, i.e., to reactions with radiolytic species of the diluent, rather than to direct interaction between radiations and ligand. In particular, the radiolysis of the organic diluents is expected to produce principally the solvated electron and its corresponding alcohol radical cation as well as neutral carbon-centered α-hydroxy radicals, according to equations reported in Table 4.^30^

**Table 4:** Main Alcohol Degradation Byproducts

equations
[RCH_2_OH] + γ → [RCH_2_OH]*
[RCH_2_OH]* → e_solv_^–^ + [RCH_2_OH]^·+^
[RCH_2_OH]^·+^ → RC·HOH + H^+^

All these radicals could interact with the extractant molecules. In addition to 1-octanol radicals, it is known from the literature that the presence of kerosene enhances solvent degradation, probably due to the formation of additional reactive species from its degradation.^30^ Besides radiolysis of organic solvent, even nitric acid can undergo degradation. In particular, the principal radiolytic species expected in the presence of oxygen and nitric acid are NO_3_^•^, NO_2_^•^, and NO_2_^-^, in addition to water byproducts (H^•^, HO^•^, HO_2_^•^, and H_2_O_2_). Among all species generated by radiolytic degradation of aqueous solutions, hydroxyl radicals (HO^•^) are the most reactive hydrogen abstracting agents since hydrogen radicals (H^•^) are indeed quickly converted to the less reactive HO_2_^•^ radicals. As a consequence of hydrogen atom abstraction from a C–H bond of the lateral chain, the proposed degradation path involves the addition of radical species coming from diluent radiolysis. Considering all these features, the hypothesized structures of some of the byproducts are reported in Table 5.

**Table 5:**
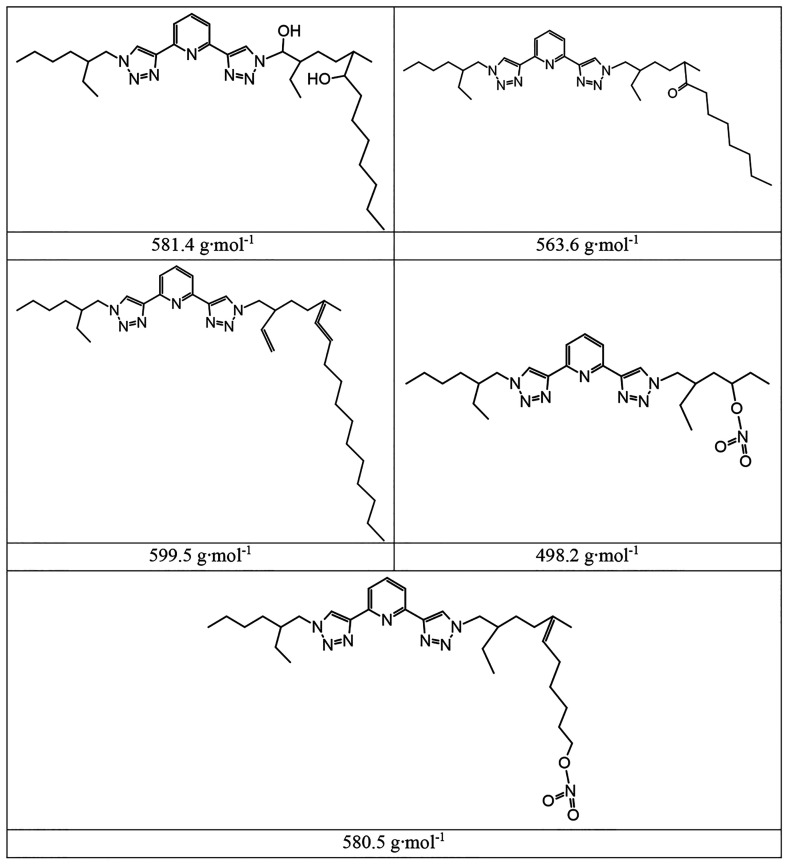
Hypothesized Structures of Identified PTEH Byproducts

The ESI-MS^2^ acquisition allowed confirmation of some of the above hypothesized species (see Figures S2–S4 and Tables S2–S4 in the Supporting Information). As reported, in all the hypothesized byproduct structures, the complexing site is preserved. Therefore, it is reasonable that the affinity toward MA of aged and irradiated solutions is preserved with respect to fresh ones, in agreement with extraction tests. Moreover, the lipophilicity seems to be not compromised. Consequently, the organic solvent selectivity for MA should be unaltered, since the complexes involving **PTEH** byproducts are not expected to migrate to the aqueous phase.

### 3.7 Back-Extraction Capability

Owing to the exceptional hydrolytic and radiolytic stability manifested by **PTEH** based solvent, back-extraction tests were performed on fresh, aged, and irradiated loaded **PTEH** solutions. As reported in Table 6, batch liquid–liquid extraction tests showed that a solution of 0.1 M nitric acid is able to strip between 99.3% ÷ 99.9% and 89.7 ÷ 96.4% of the complexed Am and Eu cations, respectively, in a single back-extraction step. Moreover, it seems that the presence of degradation byproducts did not influence the cation release.

**Table 6:** **R**esults of Back-Extraction Tests Performed Contacting 0.1 M Nitric Acid Solution with the Following **PTEH** Loaded Solutions: (i) Fresh, (ii) Aged for 37 Days, (iii) Aged for 37 Days in Contact with 3 M Nitric Acid, (iv) Irradiated at 200 kGy, (v) Irradiated at 300 kGy, and (vi) Irradiated at 200 kGy in Contact with 3 M Nitric Acid

	release %	
test	Am	Eu
i	99.3%	89.7%
ii	99.9%	91.8%
iii	99.8%	95.0%
iv	99.7%	96.4%
v	99.6%	94.9%
vi	99.6%	90.8%

Those results are promising for fostering the recyclability of **PTEH** solvent and the implementation of subsequent MA purification and conversion steps.

## 4 Conclusions

The extraction properties of **PTEH** were extensively studied in this work. Its promising MA selectivity was demonstrated even in the presence of aqueous feed containing real waste concentrations of Y and lighter Ln. Furthermore, its extraction kinetics was verified to be sufficiently fast for implementation in industrial equipment without the necessity to add phase-transfer catalysts, contrarily to the reference CyMe_4_-BTBP ligand. The effect of temperature on **PTEH** performances was successfully assessed, even if a slightly decreasing trend of the Am(III)/Eu(III) *SF* was highlighted. Furthermore, the solvent stability toward aging, hydrolysis, and gamma irradiation up to 300 kGy was positively demonstrated, and the main degradation byproducts were hypothesized. Moreover, it was proved that diluted nitric acid solutions are suitable for the full release of the complexed cations from the loaded organic phases, thus allowing feasible solvent recycling.

In conclusion, thanks to the **PTEH** promising extracting properties, the scientists on the committee of the European GENIORS project have promoted **PTEH** as a concrete alternative to the reference CyMe_4_-BTBP ligand. Therefore, the **PTEH** ligand is worth being further studied in view of ascertaining its applicability to the *1-c-*SANEX process. As a way of example, the well-known CyMe_4_-BTBP issues with some fission and corrosion products (e.g., Mo, Zr, Pd, Ag) have been solved by the addition of washing steps, masking agents, etc., but the management of some other elements (e.g., Cd, Ni, Mn) still remains problematic. In order to design a process as simple and effective as possible and limit the amount of secondary waste produced, the influence of such fission and corrosion products on **PTEH** extraction are under investigation. Moreover, CyMe_4_-BTBP is characterized by limited loading capability which hindered its application on high metal loaded fuels, and so, the assessment of **PTEH** behavior in the presence of high metal concentration could clarify if it is able to overcome this issue. Additionaly, even **PTEH** capability to extract TRansUranium (TRU) elements in all oxidation states will be investigated with a view to be employed in GANEX (Grouped ActiNide EXtraction)-like processes. Finally, additional activities aimed at clarifying the complexation mechanism and the observed degradation byproducts are ongoing.

## Supporting Information Available

Separation factors obtained between Am and lighter lanthanides and details about identification of degradation byproducts (PDF)
