# Supplementary material for: Promising Lipophilic PyTri Extractant for Selective Trivalent Actinide Separation from High Active Raffinate
Source: Ind Eng Chem Res. 2022 Mar 21;61(12):4436–44. doi: 10.1021/acs.iecr.2c00104 (PMC8972958; doi:10.1021/acs.iecr.2c00104)
Supplement: Supplementary file 1 — ie2c00104_si_001.pdf [file ie2c00104_si_001.pdf]

Supporting Information

Promising lipophilic PyTri extractant for selective trivalent actinide separation from High Active Raffinate

Annalisa Ossola[1], Eros Mossini[1], Elena Macerata[1], Walter Panzeri[2], Andrea Mele[2,3], Mario Mariani[1]*

[1] Department of Energy, Politecnico di Milano, Piazza L. da Vinci 32, I-20133 Milano, Italy; Tel: +39.02.23996385;

[2] C.N.R. – Consiglio Nazionale delle Ricerche, Istituto di Scienze e Tecnologie Chimiche "G. Natta" (SCITEC), Sezione "U.O.S. Milano Politecnico", Milan, Italy

[3] Department of Chemistry, Materials and Chemical Engineering "G. Natta", Politecnico di Milano, Piazza L. da Vinci, 32, 20133 Milano, Italy

* Corresponding author:

Tel.: +39 02 2399 6358; fax: +39 02 2399 3863.

e-mail address: elena.macerata@polimi.it.

Table of Contents

1. Selectivity towards Ln 3
2. Resistance towards hydrolysis and radiolysis 3
2. By-products identification 4

1. Selectivity towards Ln

Table S1 Separation factors of ^{241}Am over trivalent ^{152}Eu, Y and lighter Ln (La-Gd) as a function of the nitric acid concentration of the aqueous phase. Organic phase: 0.2 M PTEH in kerosene + 10 vol.% 1-octanol mixture. Aqueous phase: HNO_3 solutions loaded with Y and lighter Ln (La-Gd), besides trivalent ^{241}Am and ^{152}Eu as radiotracers

	SF(Am^{3+}/M^{3+})		
	**1 M**	**2 M**	**3 M**
**Y**	524 ± 74	321 ± 45	213 ± 30
**La**	> 1000	300 ± 42	128 ± 18
**Ce**	724 ± 102	330 ± 46	165 ± 23
**Pr**	378 ± 53	375 ± 53	237 ± 33
**Nd**	239 ± 33	378 ± 53	340 ± 48
**Sm**	132 ± 18	196 ± 27	210 ± 29
**Eu**	99 ± 14	110 ± 15	102 ± 14
**Gd**	91 ± 12	76 ± 10	66 ± 9
**^{152}Eu**	82 ± 11	86 ± 12	79 ± 11

2. Resistance towards hydrolysis and radiolysis

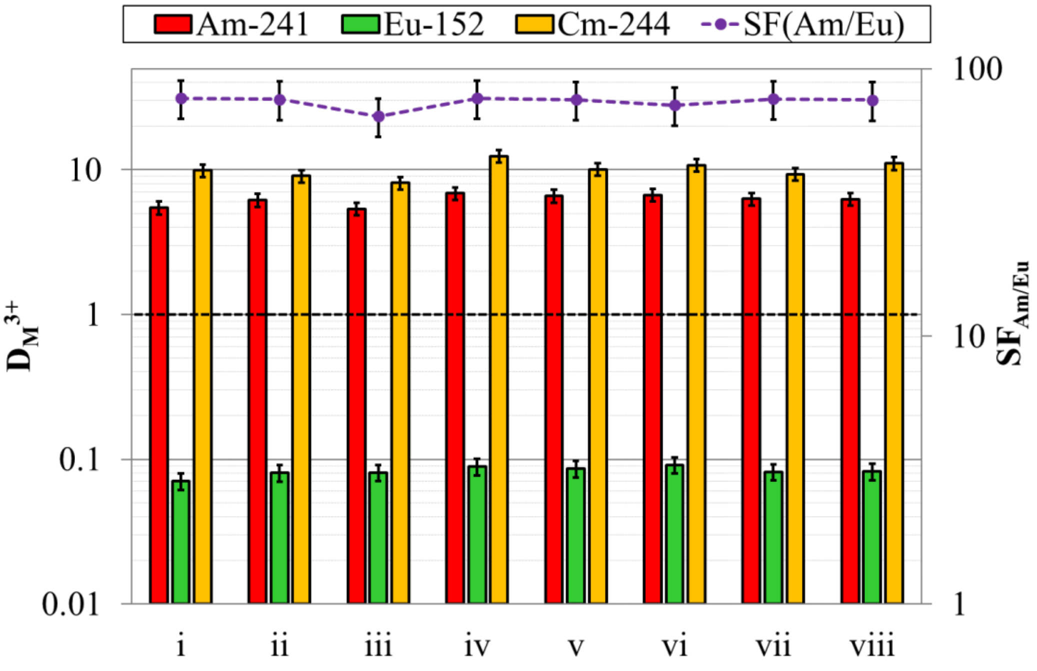

Figure S1 Distribution ratios and separation factors of trivalent ^{241}Am, ^{244}Cm and ^{152}Eu obtained by contacting 3 M HNO_3 solutions spiked with trivalent ^{241}Am, ^{244}Cm and ^{152}Eu (ca. 10 kBq/mL each) with the following PTEH solutions: (i) fresh, (ii) aged for 71 days, (iii) aged for 169 days in contact with 3 M nitric acid, (iv) irradiated at

100 kGy, (v) irradiated at 200 kGy, (vi) irradiated at 300 kGy, (vii) irradiated at 100 kGy in contact with 3 M nitric acid and (viii) irradiated at 200 kGy in contact with 3 M nitric acid

2. By-products identification

The ESI-MS^2 spectrum of the protonated adduct of the by-product with molar mass 563.6 g·mol^{-1} and identification attempts of some fragments are reported in Figure S2 and in Table S2, respectively. It was hypothesized to be the outcome of 1-octanal radical addition on the lateral chain. 1-octanal is supposed to be produced by secondary reaction of 1-octanol by-product. The ESI-tandem mass spectrometry attested that the proposed structure for by-product with molar mass 563.6 g·mol^{-1} is reasonable.

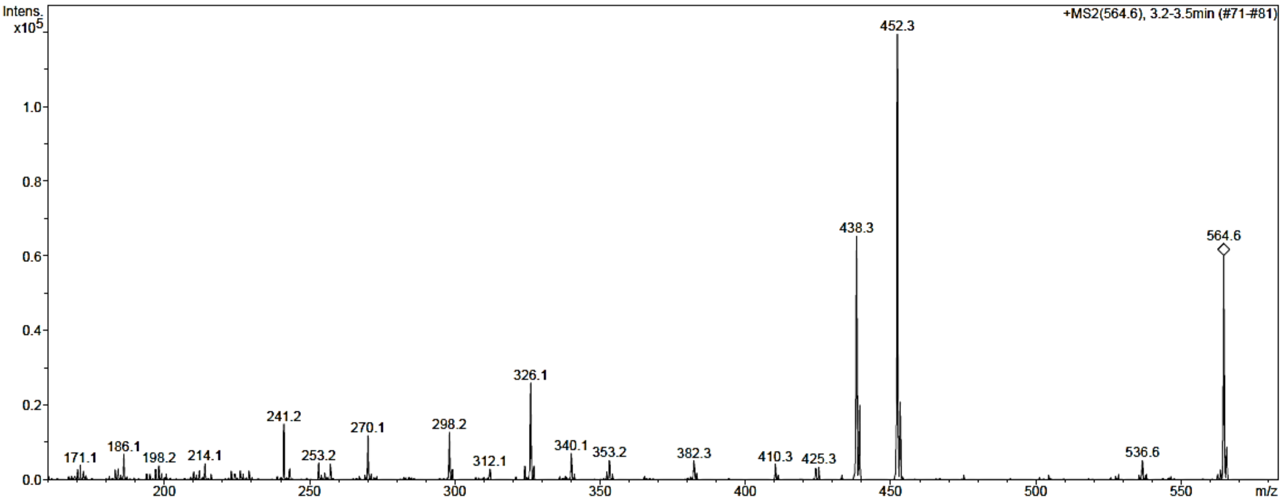

Figure S2 HPLC coupled with ESI-MS^2 spectrum of protonated adduct of PTEH by-product with molar mass 563.6 g·mol^{-1}

Table S2 Attempt of identification of some fragments in the MS^2 spectrum of PTEH by-product with molar mass 563.6 g·mol1

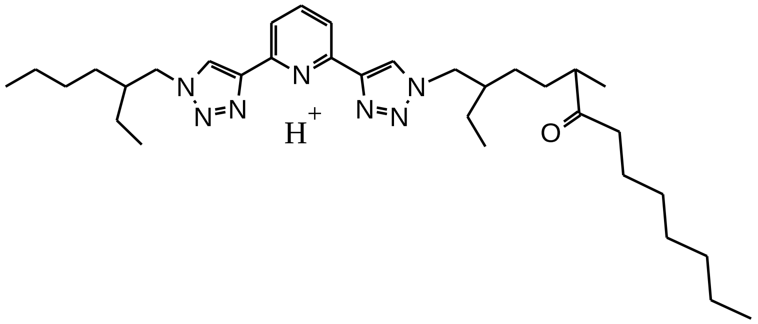
***m/z* = 564.8**
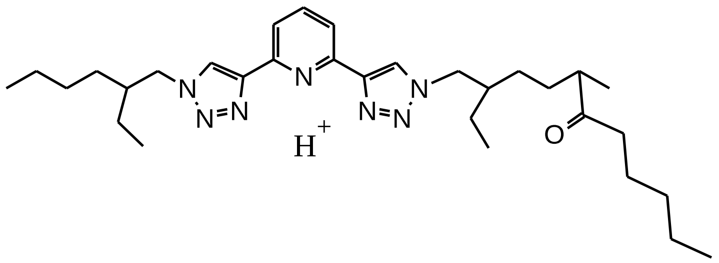
***m/z* = 536.7**

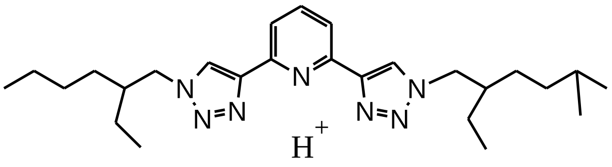
***m/z* = 452.6**
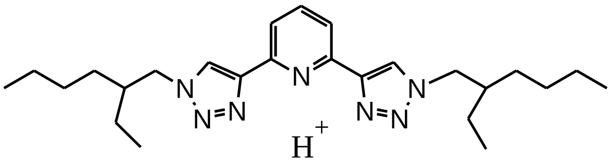
***m/z* = 438.6**
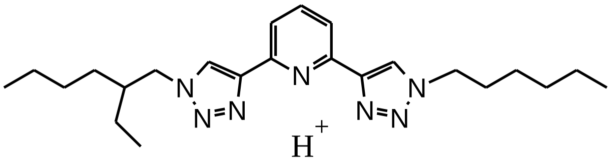
***m/z* = 410.6**
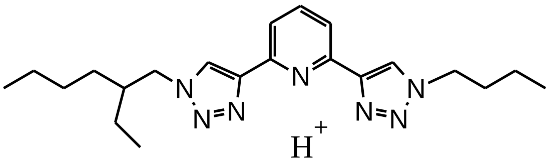
***m/z* = 382.5**
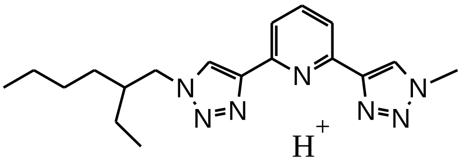
***m/z* = 340.4**
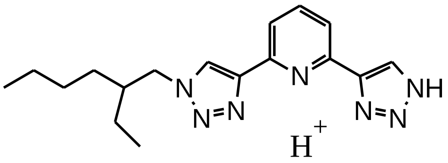
***m/z* = 326.4**
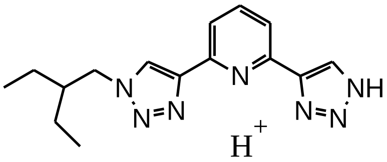
***m/z* = 298.4**
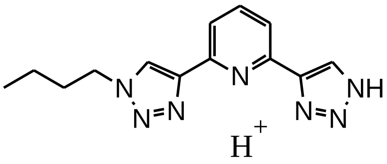
***m/z* = 270.3**

The MS^2 spectrum of the protonated adduct of the by-product with molar mass 599.5 g·mol^{-1} and identification attempts of some fragments are reported in Figure S3 and in Table S3, respectively. It was hypothesized that a kerosene carbon-centered radical is added on the lateral chain. As in the previous case, the ESI-tandem mass spectrometry attested that the proposed structure for by-product with molar mass 599.5 g·mol^{-1} is reasonable.

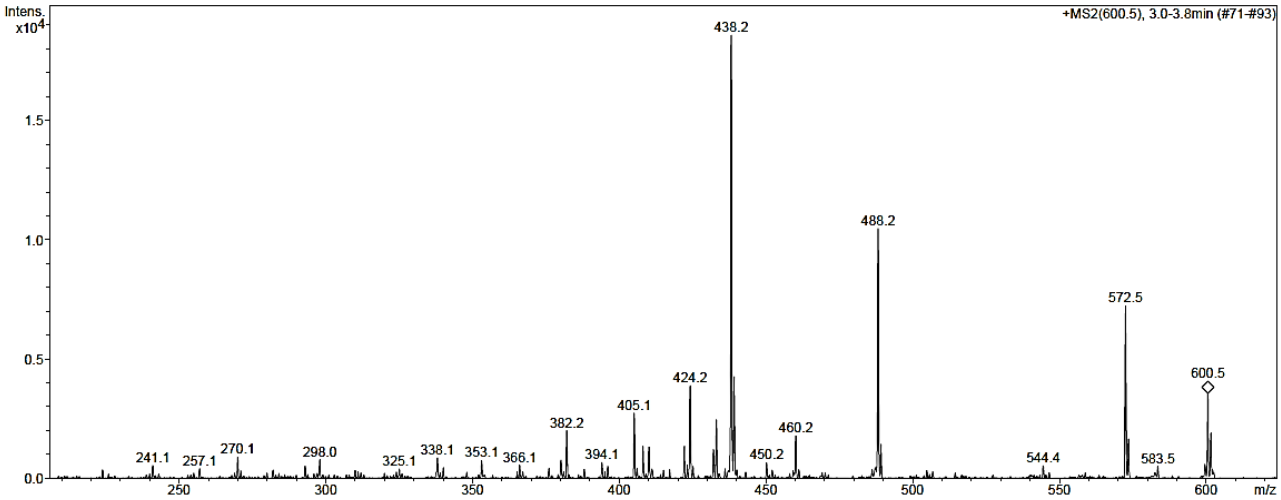

Figure S3 HPLC coupled with ESI-MS^2 spectrum of protonated adduct of PTEH by-product with molar mass 599.5 g·mol^{-1}

Table S3 Attempt of identification of some fragments in the MS^2 spectrum of PTEH by-product with molar mass 599.5 g·mol^{-1}

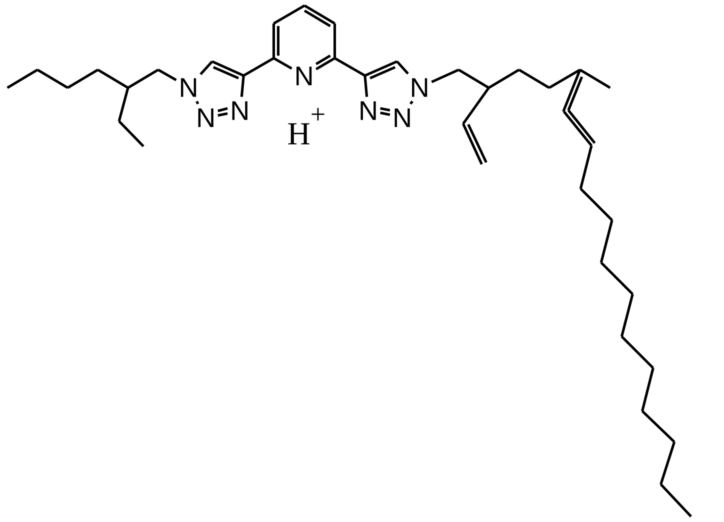
***m/z* = 600.9**

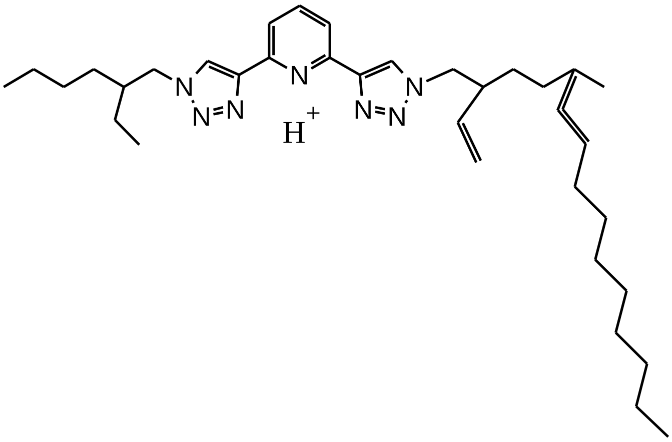

***m/z* = 572.8**

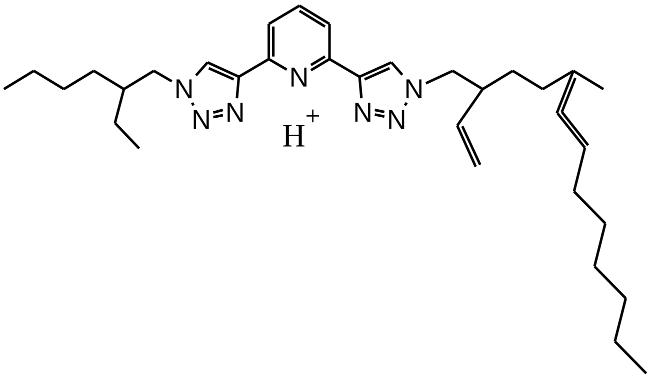

***m/z* = 544.8**

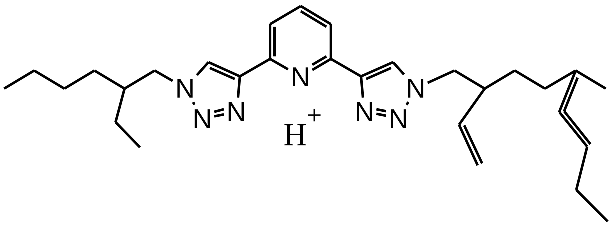

***m/z* = 488.7**

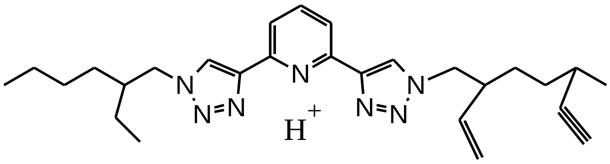

***m/z* = 460.6**

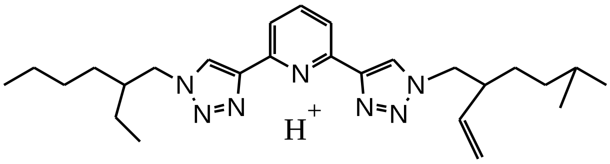

***m/z* = 450.6**

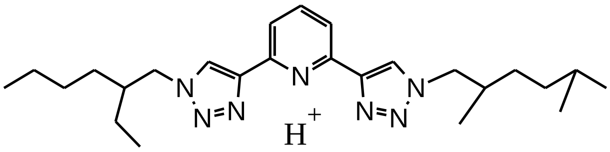

***m/z* = 438.6**

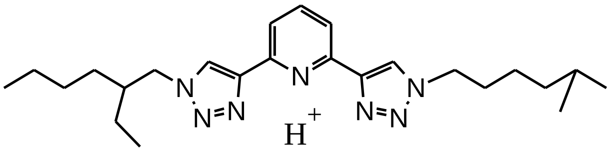
***m/z* = 424.6**
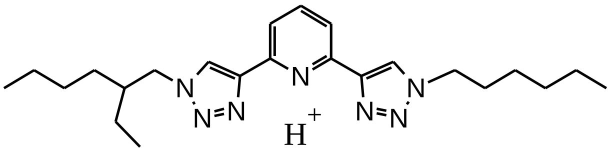
***m/z* = 410.6**
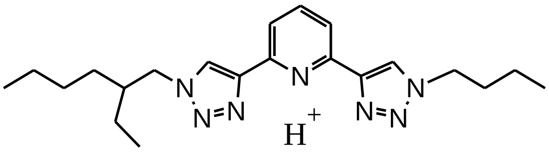
***m/z* = 382.5**

Finally, the MS^2 spectrum of the protonated adduct of the by-product with molar mass 580.5 g·mol^{-1} and identification attempts of some fragments are reported in Figure S4 and in Table S4, respectively. In this case, the addition of C_6 and nitric acid radicals produced an adduct on the lateral chain. Once again, the ESI-tandem mass spectrometry proved that the proposed structure for by-product with molar mass 580.5 g·mol^{-1} is realistic.

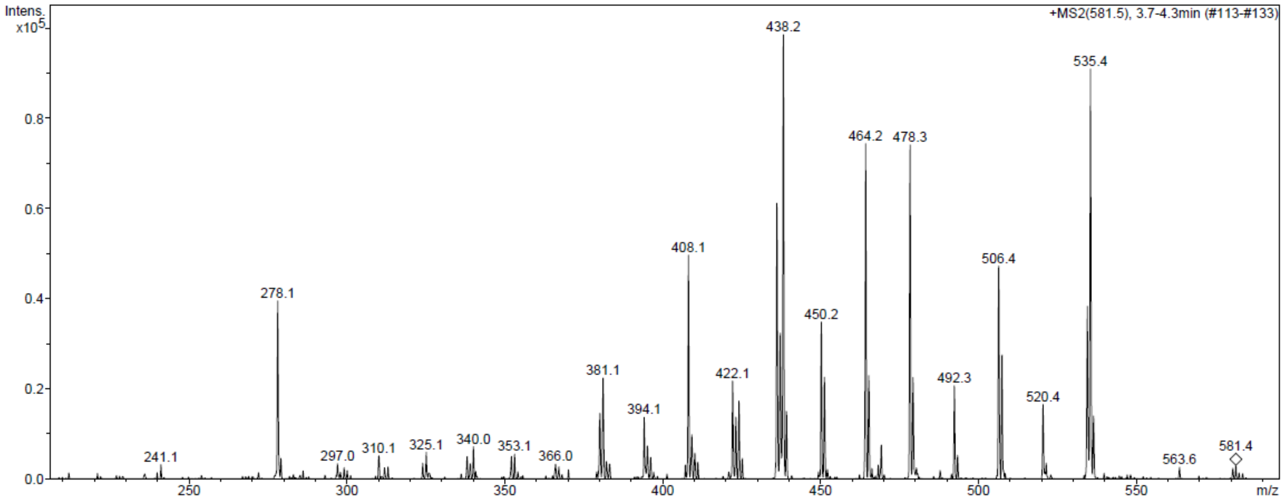

Figure S4 HPLC coupled with ESI-MS^2 spectrum of protonated adduct of PTEH by-product with molar mass 580.5 g·mol^{-1}

Table S4 Attempt of identification of some fragments in the MS^2 spectrum of PTEH by-product with molar mass 580.5 g·mol^{-1}

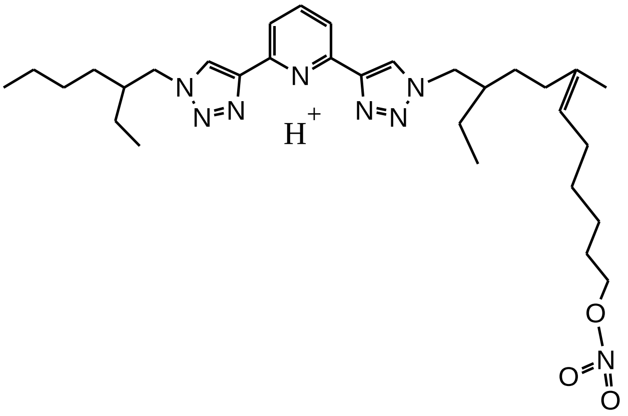
***m/z* = 581.7**
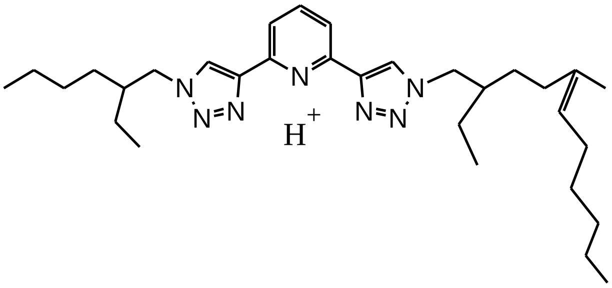
***m/z* = 520.7**
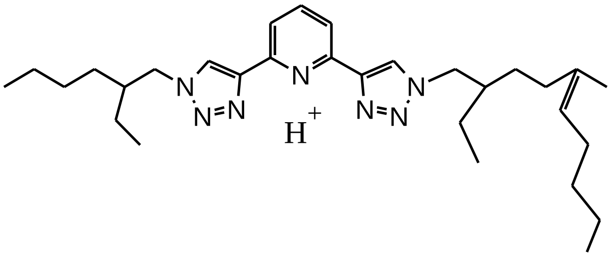
***m/z* = 506.4**
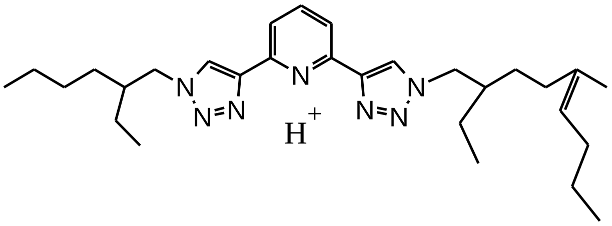
***m/z* = 492.7**
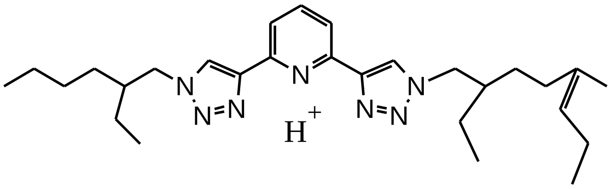
***m/z* = 478.7**
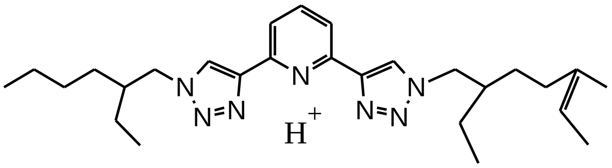
***m/z* = 464.2**

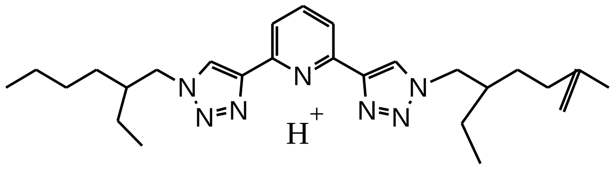

***m/z* = 450.6**

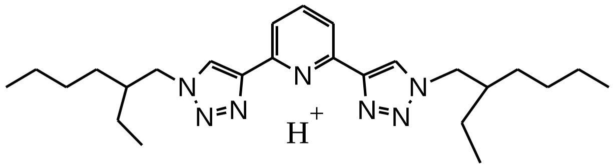

***m/z* = 438.6**

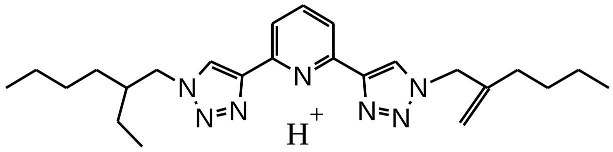

***m/z* = 422.6**

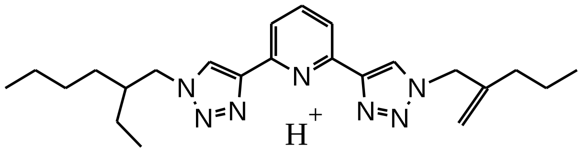

***m/z* = 408.6**

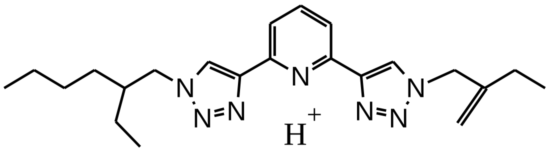

***m/z* = 394.5**
